# Risk prediction models of primary graft dysfunction in cardiac transplant patients: a need to improve?

**DOI:** 10.3389/fcvm.2024.1478821

**Published:** 2024-09-23

**Authors:** Chloe Grzyb, Dongping Du, Balakrishnan Mahesh, Nandini Nair

**Affiliations:** ^1^ College of Medicine, The Pennsylvania State University, Hershey, PA, United States; ^2^ Industrial, Manufacturing, Systems Engineering, Texas Tech University, Lubbock, TX, United States

**Keywords:** PGD, cardiac transplant, risk factor prediction, DCD, DBD

## Introduction

Cardiac transplant remains the gold standard for end-stage heart failure. Over 5,000 patients now undergo transplants each year (1). The leading cause of 30-day mortality after transplant is primary graft dysfunction (PGD). The incidence of PGD is currently better defined after the definition has been established and a recent meta-analysis found pooled incidence of PGD between 1.6% and 7.7% (2–5). The prevalence of PGD has increased, as reported in a 2024 update from the International Consortium on PGD (6, 7). PGD is currently defined as ventricular dysfunction of a donor graft that fails to provide hemodynamic stability within 24-hour post-transplantation that cannot be attributed to any other cause (3, 5). Secondary causes may include graft dysfunction due to pulmonary hypertension, intraoperative complications, or hyperacute rejection. It can be separated into PGD-LV, for disease affecting the LV or biventricular failure, and PGD-RV, when due to isolated RV involvement (5).

The 2014 consensus statement by Kobashigawa et al. based on the modified Delphi method provided a definitive definition and grading system that helped define the true incidence of PGD (5). This consensus supported the use of inotropes such as phosphodiesterase-3 inhibitors and catecholamines for initial management and helped to identify risk factors which underlie PGD. Therapy can be escalated to use intra-aortic balloon pumps followed by initiation of mechanical circulatory support (MCS) and ultimately extracorporeal membranous oxygenation (ECMO) (5). Plasmapheresis may be used to combat inflammatory cytokines that are thought to underlie PGD (5, 8). The gold standard for preventing PGD is a cooled flush of preservation fluid for myocardial protection, which helps improve tolerance to ischemic time (9).

In this opinion paper, we discuss risk factors and linear and machine learning models used to predict outcomes of PGD and list possible strategies to improve the discriminatory power of the risk prediction models for PGD.

### Risk factors

Common risk factors for PGD have been grouped into donor factors, recipient factors, and procedural factors (9).

### Donor factors

Age has been identified as a significant risk factor, possibly due to decreased tolerance for long ischemic times in the hearts of older patients (10). Singh et al. identified an odds risk of 20% for each decade increase in age (9). Another risk factor of PGD is the cause of donor death. PGD rates were increased in patients who died of intracranial hemorrhage compared to traumatic death, which may be attributed to a catecholamine surge decreasing myocardial function (11). Gender mismatch between donor and recipient may be a risk factor for PGD and had a worse survival at five years, which persisted despite size-matching organs. Left ventricular hypertrophy of donor hearts should be kept under 14 mm and without associated EKG changes (12). Hearts with LVH are more sensitive to ischemic changes due to supply/demand mismatches. LVH in the setting of long ischemic times and older age shows increased mortality (13, 14). Donor ionotropic requirements are shown to induce LV and RV dysfunction and are risk factors for PGD (15–17). Most recently donor hyperoxia (donor supported on FiO2 ≥ 40%) at recovery has been shown to be a novel risk factor for severe primary graft dysfunction and early death of the recipient (18).

### Recipient factors

The need for pre-operative MCS is strongly linked to the development of PGD which is possibly linked to the activation of inflammatory mediators, causing vasodilation and lowering of systemic vascular resistance (8, 19, 20). Increased ischemic time may underly the increased incidence of PGD in MCS patients due to a summative effect of blood exposure to the surfaces of bypass machines which can further exacerbate the inflammatory response (20).

Pre-operative recipient amiodarone is an indicator of the critical, pro-arrhythmic state of these patients. Dose and duration-dependent relationships between amiodarone use and PGD post-transplant have been identified (21, 22). However, early studies have also found that patients receiving pre-operative amiodarone had lower post-operative heart rates that were more likely to require atrial pacing without an increase in postoperative mortality (23).

Other notable recipient factors affecting PGD were diabetes mellitus, age, and re-sternotomy (24, 25). Advanced glycation end products and coronary endothelial inflammation may also induce graft loss. Diabetes has been identified as a predictor of graft loss within and after the first year of transplant. Advanced recipient age is associated with PGD and mortality, likely due to increased comorbidities and increased rates of inflammation (17, 26, 27). Prior recipient sternotomy from congenital surgery, CAD implantation, or CABG presents a challenging dissection of adhesions during transplantation and thus may increase ischemic times or increase the risk for reoperation and bleeding. It has been linked to a three-fold increase in risk of PGD (25). Heart size discrepancies predict mortality at 30 days and one year, likely due to insufficient cardiac index to support body habitus (28).

### Procedural factors

Prolonged ischemic time during transport and surgery increases the risk of PGD (24–27, 29). Warm ischemic time refers to surgical periods or aortic clamping where blood flow is halted, while cold ischemic time refers to time spent in cold storage. Cardiopulmonary bypass time is linked to PGD due to the occurrence of ischemic reperfusion injury as well as systemic inflammatory pathway activation (30).

### Current risk prediction models for PGD and their limitations

Few risk prediction models exist in the current literature derived using linear regression and machine-learning algorithms.

### Linear models

The prediction models for PGD derived using linear regression are RADIAL, PREDICTA, and ABCE (24, 31, 32). RADIAL was developed using a Spanish cohort of transplant patients to help establish a definition for PGD as well as a predictive score (24). This was a single-center retrospective study of 621 patients. They found six multivariate risk factors of PGD: Right atrial pressure ≥ 10 mm Hg, recipient Age ≥ 60 years, Diabetes mellitus, Inotrope dependence, donor Age ≥ 30 years, and Length of ischemic time >240 minutes. The c-statistic between the actual and predicted PGD incidence was 0.547, demonstrating reasonable predictive ability using the multivariable (stepwise- backwards elimination) logistic regression methodology. However, the limitations included a small cohort from a single center, and the definition of PGD was not universal at the time it was proposed. Hence this model does not appear to perform well when applied to severe PGD in the current day scenarios (7).

PREDICTA was developed using data from 613 patients between 10/2012 and 9/2016 at six UK transplant centers. A multivariate logistic regression approach was used and compared to the RADIAL score (31). The c-statistic was 0.704 compared to 0.547 from the RADIAL score. The risk factors identified in this cohort also included diabetes and increasing donor age. Unlike the RADIAL score, they also identified preoperative MCS, prolonged cardiopulmonary bypass time, and prolonged implant time. The incidence of PGD was 38%. Though this model had a multicenter cohort it also had its share of limitations in that it still had a small number of patients limited to the UK hospitals and lacked external validation in an international setting.

ABCE risk score was based on the severity of the disease (32). This was a single-center retrospective study that included 734 patients between 10/2012 and 9/2016. Different risk factors were identified for mild to moderate PGD vs. severe PGD which may suggest different mechanisms of disease (32). Multivariable logistic regression was performed for mild/moderate and machine learning was used for severe PGD patients.

PGD occurred in 24% of the cohort. Within the PGD group, 42% developed mild PGD, 33% developed moderate PGD, and 25% developed severe PGD. Prior cardiac surgery, recipient GDMT (ACEI/ARB/ARNI plus MRA), treatment with amiodarone plus a beta blocker, and ischemic time were identified as four recipient risk factors for PGD (32). In addition, 3 surgical factors such as prolonged ischemic time, more RBC transfusions, and more platelet transfusions were associated with PGD. Using machine learning with a nested cross-validation scheme for severe PGD an AUC of 0.79 and 0.77 were obtained for the training and validation sets respectively. This model has a c-statistic of 0.77 compared to 0.41 obtained with the RADIAL score for severe PGD. Only 48% of patients with severe PGD survived one year, while mild/moderate PGD did not affect survival. ABCE risk score has its merits in that it showed for the first time that risk factors for mild/moderate and severe PGD are varied as well as shared and that only severe PGD impacted mortality. Additionally, this model used a machine learning approach for risk prediction of severe PGD. However, it is still limited by its small cohort size based on a single center lacking robust external validation.

### Machine learning models

Two models using machine learning algorithms exclusively exist in the current literature Linse et al. developed a non-linear artificial neural networks (ANN) model to evaluate donor-recipient variables for death due to PGD at 30 days post-transplantation using a cohort of 64,964 patients using the ISHLT registry (33). The incidence of PGD at 30 days was 3.7%. Thirty-three of 77 risk variables were identified as relevant. The model had a c-score of 0.70 (95% CI: 0.68–0.72) compared with the RADIAL score which had a c-statistic of 0.53 (CI 0.52, 0.54). The most influential variables were underlying heart failure diagnosis, ischemia time, and sex mismatch, which were not among the international heart transplant survival algorithm (ISHTA) (34) and renal function had a lower impact. 90% of the variables had missing data with a mean of 42%. The limitation of this model is that despite its generalizability, it uses registry data that contains missing and misclassified data which can introduce bias despite multiple imputations even in the setting of cross-validation and cross-testing. This model also has a focus on short-term outcomes at 30 days.

In 2022, an international, multicenter PGD Consortium was formed to redefine the clinical risks of PGD (7). In this cohort, 2746 patients were enrolled since 2015. Of these, 73.4% were from North America and 26.6% were from Europe. 7.8% had severe PGD. The radial score when applied to this cohort showed a suboptimal discriminatory power of 0.53. Multivariate logistic regression applied to this cohort identified 3 risk factors acute—preoperative dialysis, durable LVAD support, and total ischemic time.

To the international PGD consortium, an ML risk-scoring algorithm was applied which included 18 variables with a c-stat of 0.729 in a training set (35). This model is limited because of the small cohort size and is pending validation (35).

## Discussion

PGD is affected by numerous variables and pathophysiology remains poorly understood. Therefore, a critical need exists for high-performing risk prediction models. More recent models have begun to use machine learning algorithms in place of traditional, linear statistical methods. Machine learning approaches may allow for a better fit between variables and outcomes by identifying patterns in datasets, especially in large, complete datasets. RADIAL, PREDICTA, and ABCE scores, which used linear regression may therefore be unable to account for complex relationships between risk factors as well as between risk factors and outcomes (24, 31, 32). However, in the existing literature machine learning models have failed to outperform linear prediction models possibly because ML algorithms operate as black boxes and may require a collective improved comprehension of how they work in small and large data sets.

Most of the existing models for PGD prediction are derived from small single-center cohorts with marginal external validation. Risk prediction models have the potential to determine candidacy for heart transplants through prediction of post-transplant outcomes and therefore play an important role in patient selection. This opinion article discusses the existing models and their limitations to highlight the knowledge gap.

### Future directions

A need exists for high-powered predictive models that can integrate many variables from multi-institutional data. This is necessary to account for heterogeneity in patient characteristics that affect patient outcomes and survival in cardiac transplant patients and hence will contribute to improving generalizability. The main barrier to improved risk prediction is complete and accurate granular data from representative populations. Missing data is another limitation, as it allows for selection bias and difficulty in quantifying variables. The use of multicenter data can introduce heterogeneity which may influence post-operative management.

The ISHLT consensus developed a standardized definition for PGD in 2014 (5). For this reason, variables identified in models developed before 2014 may be inaccurate in predicting risk in the contemporary era.

**Figure 1:**
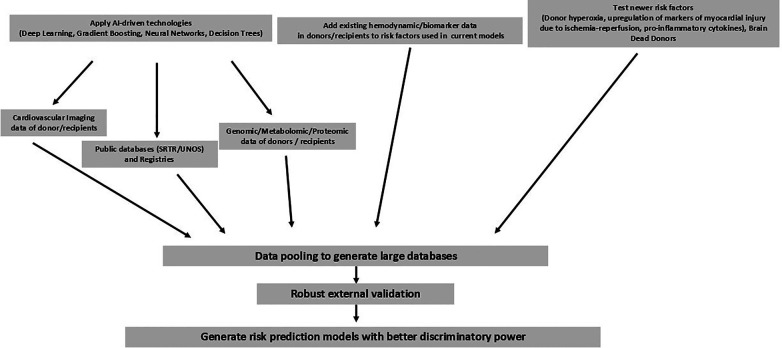
Suggested strategies to improve risk models for PGDin cardiac transplantation.

The use of pre-processed data may affect the performance of models, as deep networks operate best with raw data where the algorithm can identify the relationships between the data (32). Figure 1 summarizes the possible strategies that can be used to improve discriminatory power. The generation of large databases and using AI-driven technologies (Deep Learning, Gradient Boosting, Neural Networks, and Decision Trees) on these databases should help iron out complex interactions between variables. The addition of new risk factors such as donor hyperoxia, brain death, and biomarkers of inflammation/myocardial injury may help increase discriminatory power.

The role of DCD (Donation after circulatory death) and DBD (Donation after brain death) hearts in predisposing to PGD is poorly understood at his time. Hence more research is needed to establish the differences between them. Additionally using different procurement methods such as normothermic regional perfusion vs. direct procurement and perfusion using the Organ Care System can impact PGD. Though severe PGD is noted with DCD hearts the length of stay and recovery is much better with DCD than with DBD suggesting different patterns of recovery depending on the cause of death. Hence DBD and DCD may appear to be risk factors with varying impacts on PGD (36) Therefore DBD and DCD can be added to the panel of risk factors to assess their role in the prediction of PGD.

## Conclusions

PGD remains the leading cause of early mortality following cardiac transplant. Its risk factors are multifactorial and require improved prediction models to improve outcomes. Limitations of current models are missing data, uneven distribution of variables, small patient cohorts and lack of robust external validation datasets. Improvement in the discriminatory ability is necessary before current models can be used to assist in clinical decision-making effectively. Prospective data collection to generate large databases and validation of results using independent data sets remain prerequisites for developing better risk prediction models.
